# Mindful of yourself: the initial evaluation of an online health promotion programme for university students

**DOI:** 10.3389/fpsyg.2026.1842551

**Published:** 2026-07-20

**Authors:** Brigitte Jenull, Janik Wiedenhöfer, Anna Resch, Linda Maurer

**Affiliations:** Department of Health Psychology, University of Klagenfurt, Klagenfurt, Austria

**Keywords:** mindfulness-based interventions (MBIs), online health promotion, self-care, self-compassion, university students

## Abstract

**Aim:**

As students are vulnerable to mental disorders, interventions for promoting mental health and well-being are urgently needed. Third-wave behavioural therapy programmes (e.g., ACT and MSC) have shown promising results in addressing these issues. The online health promotion programme SAM was developed in a multistage process using a participatory design with students at the University of Klagenfurt, Austria. The name SAM was derived from the German terms for the three main components of programme content: self-care (*S*elbstfürsorge), mindfulness (*A*chtsamkeit), and self-compassion (*M*itgefühl). It was hypothesised that SAM would (1) increase students’ mindfulness, self-compassion, and well-being and (2) decrease levels of depression, anxiety, and stress.

**Subject and methods:**

Participants were randomly assigned to either the SAM (*n* = 53) or the low-dose educational group (*n* = 65), both receiving basic content on nutrition, exercise, relaxation, and stress reduction. Students in the SAM group received a self-administered 21-day intervention, and outcomes were assessed using questionnaires.

**Results:**

A total of 118 students participated in both the baseline assessment and postassessment. A mixed-model MANOVA yielded nonsignificant interaction and group effects but a significant time effect in the expected direction for most outcomes. Across both conditions, participants showed improvements in well-being, and self-compassion as well as decreases in depression, anxiety, and stress symptoms at postassessment. Whilst no significant group-level time effect was observed for mindfulness, Reliable Change Index (RCI) analyses indicated clinically meaningful improvement in mindfulness for 13.6% of participants. The highest rates of reliable improvement were found for well-being (21.2%) and self-compassion (20.3%). However, RCI analyses also identified clinically meaningful deterioration in a subset of participants, particularly for depression (19.5%) and stress (13.7%), highlighting substantial heterogeneity in individual responses despite relatively modest changes at the group level.

**Conclusion:**

Although no differential effects of the SAM programme were observed, the findings highlight the potential impact of small interventions and offer valuable insights for future research. However, the SAM programme could benefit from these insights and is currently under revision.

## Introduction

Previous studies have demonstrated that students are vulnerable to mental disorders and mental health conditions. Almost two-thirds of college students reported a 12-month DSM disorder (Mason et al., 2025), with a high prevalence of depression and anxiety symptoms globally (Ibrahim et al., 2013; Li et al., 2022; Paiva et al., 2025). These conditions were associated with severe role impairments at the university (Alonso et al., 2018). They increased the odds of low levels of mental well-being (Navarra-Ventura et al., 2024), which is more than just the absence of psychiatric disease—it represents a combination of feeling good and functioning well in a psychological sense (Huppert, 2009). In addition, perceived stress was found to have a strong positive association with mental disorders amongst students, and stressors were found to have an additive effect, indicating that multiple sources of stress increase the likelihood of mental disorders (Karyotaki et al., 2020). This particularly highlights the detrimental impact of academic stress and difficulties (Li et al., 2022). Despite these risk factors and far-reaching consequences, only a minority of students received adequate treatment (Auerbach et al., 2016), indicating the need for quick, accessible student-tailored prevention and intervention programmes (Alonso et al., 2018; Li et al., 2022). Given the fundamental changes in health care systems due to the COVID-19 pandemic, particularly with respect to the establishment of alternatives to face-to-face treatments, digital interventions have gained importance in maintaining post-pandemic mental health (Husain et al., 2021). The scarcity of mental health care options underscores the critical importance of freely accessible digital interventions, particularly in rural regions (Mittmann et al., 2025).

Mindfulness-based interventions (MBIs) might represent a promising approach for addressing these issues. Studies have demonstrated the effectiveness of MBIs in reducing symptomsWHO-5 of depression, anxiety, and stress (Querstret et al., 2018, 2020) whilst increasing well-being (Dawson et al., 2020; De Vibe et al., 2018; Querstret et al., 2020). Cognitive, behavioural, and mindfulness-based stress reduction interventions may also be effective for university students, as they have been shown to reduce anxiety, depression, and cortisol levels (Regehr et al., 2013). Mindfulness is “paying attention on purpose, in the present moment, and nonjudgmentally to the unfolding of experience” (Kabat-Zinn, 2003: 145). It plays a crucial role in various mental processes. For instance, it enhances self- and emotion regulation (Baer, 2010; Hölzel et al., 2011; Shapiro et al., 2006) and is a precondition of self-compassion (Neff, 2003b). Being mindful fosters the balance between the awareness of painful inner states but concurrently avoids overidentification (Neff, 2003a). This stance of nonjudgment and detachment mitigates self-criticism (Jopling, 2000), paving the way for self-kindness (Neff, 2003a). Accordingly, empirical evidence has demonstrated that MBIs increase self-compassion in nonclinical populations (Golden et al., 2021), initiating self-soothing mechanisms that contribute to affect regulation (Gilbert, 2010). Therefore, a self-compassionate attitude is considered an important self-care skill (Cook-Cottone and Guyker, 2018), and it has been shown to predict personal and professional self-care (Jay Miller et al., 2019), whilst higher levels of self-compassion have also been associated with improved mood and lower levels of depression, anxiety, and stress (Neff and Germer, 2013). Building on these developments, the online health promotion programme SAM is based on the theoretical proposition that mindfulness may serve as a core element underpinning self-compassion and self-care. Based on previous studies that have explored these relationships, we assume a relationship amongst the programme components (mindfulness, self-compassion, and self-care). In a meta-analysis on stress management, Chiesa and Serretti (2009) found that mindfulness-based training leads to positive outcomes for self-compassion. Jay Miller et al. (2019) reported a predictive relationship between self-compassion and personal and professional self-care. A student sample (Slonim et al., 2015) revealed that the relationship between self-care and well-being is stronger amongst students with higher dispositional mindfulness, indicating that mindfulness also influences the degree of self-care engagement.

SAM is an acronym for the German terms for self-care (Selbstfürsorge), mindfulness (Achtsamkeit), and self-compassion (Mitgefühl), and it has a self-administering design. Psychotherapy in this field has recently focused on the context of human experience, i.e., the functions of thoughts, feelings, and cognitions (Guercio, 2022; Hayes, 2004; Hayes and Hofmann, 2021), and the relevance of mindfulness, acceptance, values, and interpersonal interactions (e.g., Hayes et al., 2006; Linehan, 1993; Segal et al., 2002; Young, 1990). For SAM, exercises from a broad range of established treatments, such as Mindfulness-based Stress Reduction (MBSR; Kabat-Zinn, 1982), Mindfulness-Based Cognitive Therapy (MBCT; Segal et al., 2002), Acceptance and Commitment Therapy (ACT; Hayes et al., 2006), and Mindful Self-Compassion (MSC; Neff and Germer, 2021), were synergistically combined. Although digital interventions have been increasingly implemented in mental health care (Zhou et al., 2021), during the pandemic of 2020, only 2% of open-access mental health apps were research-based (Ratheesh and Alvarez-Jimenez, 2022). Emerging evidence suggests that online Mindfulness-Based Interventions (MBIs) can improve mental health outcomes, with small but significant effects on depression, anxiety, well-being, and mindfulness, and the strongest effects observed for stress reduction (Linardon et al., 2024; Spijkerman et al., 2016). SAM was developed in a multistage process using a participatory design. In collaboration with students, through several feedback and revision loops, we established the programme in its current form. For an initial evaluation of the efficacy of the current version of SAM, this study aimed to investigate whether SAM (1) increases students’ well-being, mindfulness, and self-compassion and (2) decreases levels of depression, anxiety, and stress.

## Methods

### Study design and procedure

This study was conducted with a convenience sample of university students using a randomised controlled trial (RCT) design. Initially, invitation letters were sent out via e-mail to Austrian universities, encouraging students to participate in the health promotion programme. Registration could be completed by providing contact data through an online survey by clicking a link attached to the email. The inclusion criteria were (1) enrolment at an Austrian university, (2) internet access, and (3) a valid e-mail address. Students who completed the survey were subsequently randomised into either the intervention or the control group. The randomisation process was conducted by an external person who was not involved in developing the programme or performing the study. A simple randomisation procedure using the statistical software R (R Core Team, 2023) was applied with a 1:1 allocation ratio. Participants’ blinding was not achievable, as is standard practise in behavioural intervention trials. Allocation concealment was maintained prior to randomisation by describing the study as a health promotion programme. Blinding of the research team was achieved by outsourcing programme delivery to the Department of Health Management. Data were collected pre- and post-intervention using the online survey platform Unipark. All participants provided informed consent, and the study was approved by the Institutional Review Board for Research Ethics at the University of Klagenfurt (ER-AAU, Austria, 033-2022), in accordance with the Declaration of Helsinki. Furthermore, in the consent form and the debriefing, all participants were informed about psychotherapeutic services that can be used anonymously and free of charge. In addition, the SAM programme was made available to all participants after the intervention phase.

### Interventions

Participants were assigned to either a 3-week SAM intervention (*n* = 53) or a low-dose educational content group (*n* = 65). The SAM intervention group (IG) received written instructions for the self-administration of the newly designed health promotion programme (see Supplementary Table S1). These instructions included daily exercises that should be conducted in the morning and evening. The SAM programme content was based on a wide range of programmes from the third wave of behavioural therapy (e.g., ACT, MBSR, and MSC). For instance, yoga sessions, body scans, mindful showering, meditation, and exercises, such as thinking about positive and negative habits and being a good friend to oneself, were included. The duration of each exercise ranged from 5 to 45 min. Motivational e-mails were sent to the intervention group regularly during the intervention period to promote adherence. The motivational emails varied in content; for example, after the first week, participants received messages such as “Great, you have already made it through a week—keep it up” On a weekday in between, they received messages such as “Remember to treat yourself like your best friend” (see Supplementary Table S2).

In contrast, the low-dose educational content group (CG) received one information sheet each week covering the topics of general dietary recommendations (e.g., nutritional rules), beneficial effects of exercise and relaxation (e.g., exercise in everyday life), and stress reduction (e.g., relaxation in everyday life). Nevertheless, the information provided was superficial and brief. Additionally, the low-dose educational content group did not receive motivational e-mails.

### Measures

#### Demographics

Sociodemographic data, including gender, age, nationality, and living situation, were collected.

#### Mindfulness

The German version of the Mindful Attention and Awareness Scale (MAAS; Michalak et al., 2008) was used to measure mindfulness. It is a 15-item self-report measure (e.g., “I might have a feeling and not realise it until later”) that uses a six-point Likert scale ranging from 1 = “almost always” to 6 = “almost never.” The internal consistency was satisfactory ( ω _MAAS_ = 0.87). In previous studies, both the German and English versions of MAAS have shown satisfactory psychometric properties (Brown and Ryan, 2003; Michalak et al., 2008).

#### Self-compassion

Self-compassion was measured using the German version of the Self-Compassion Scale (SCS; Hupfeld and Rufieux, 2011). This self-report questionnaire comprises 26 items (e.g., “When things go badly for me, I see this difficulty as a part of life that everyone goes through at some point”), and the response options range from 1 = “very rarely” to 5 = “very often.” This construct consists of three basic components: self-kindness, common humanity, and mindfulness. The partly negative and partly positive wording of the items results in a total of six factors. The SCS is considered a valid, flexible, and reliable method for measuring both general self-compassion and different components of the construct (Hupfeld and Rufieux, 2011). In this study, we used the general score of the SCS, and the coefficient ω was satisfactory ( ω _SCS_ = 0.92).

#### Well-being

The well-being index (WHO-5; Brähler et al., 2007) is a self-report questionnaire consisting of five items (e.g., “In the last 2 weeks, I have felt calm and relaxed”). These can be answered on a six-point Likert scale from 0 = *“*at no time” to 5 = *“*all the time” to provide information about well-being. The WHO-5 is a frequently used screening instrument with excellent psychometric properties (Allgaier et al., 2012; Brähler et al., 2007). In our study, the internal consistency was acceptable ( ω _WHO-5_ = 0.78).

#### Depression, anxiety and stress

The Depression, Anxiety and Stress Scales (DASS-21; Nilges and Essau, 2015) contain 21 items for self-assessment (e.g., “I felt anxious for no reason”) of the three subscales of depression, anxiety, and stress. Using a 3-point Likert scale (0 = *“*Did not apply to me at all” to 3 = *“*Applied to me very much or most of the time”), the self-report questionnaire focuses on the core psychological aspects of depression, anxiety, and stress. DASS-21 has been psychometrically tested in various studies and has been proven to be reliable and valid (Bibi et al., 2020; Lovibond and Lovibond, 1995; Nilges and Essau, 2015). We analysed the subscales separately; the internal consistency was satisfactory for all three subscales ( ω _DASS_dep_ = 0.88; ω _DASS_anx_ = 0.80; ω _DASS_stress_ = 0.82).

### Statistical analyses

**Figure 1:**
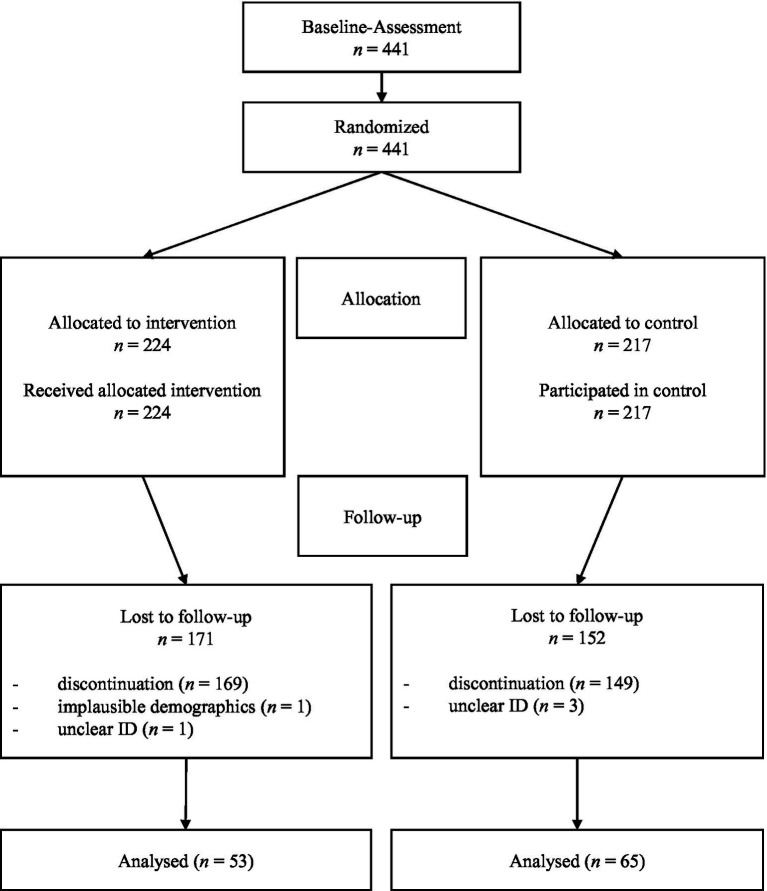
CONSORT flow diagram.

For the present study, pre- and post-intervention datasets were screened separately for noncompleters in the first step. Noncompleters were defined as participants who did not complete the survey. These participants were excluded from further analysis. Pre- and post-intervention datasets were matched together based on their identification code using SPSS and subsequently screened for doubled identification codes, unplausible matches (e.g., age increased drastically within 3 weeks), and matches that were not correctly identified automatically (e.g., capitalization in either pre- or post-intervention identification code). Participants with no matching identification code pre- or post-intervention were excluded from further analysis. Further details are provided in the CONSORT flow diagram (Figure 1).

The final dataset was then checked for multivariate outliers using the Mahalanobis distance with a cut-off of χ^2^(12) = 32.909, *p* = 0.001. Skewness and kurtosis were calculated for each item per group using the R package “psych” (Version 2.4.3; Revelle, 2024), with applied cut-off values of ±2 for skewness and ±7 for kurtosis (Curran et al., 1996). Levene’s tests were used to examine homoscedasticity between the two groups using the R package “car” (Fox and Weisberg, 2019). Moreover, baseline differences between the IG and CG in terms of the outcome variables were explored in R (R Core Team, 2023) utilising independent two-sample *t* tests. The correlations of the outcome variables are presented in Table 1.

**Table 1:** Correlations of outcome variables of the baseline assessment.

Variable	1	2	3	4	5	6
1. MAAS	–					
2. SCS	0.369	–				
3. WHO-5	0.343	0.575	–			
4. DASS_dep	−0.483	−0.538	−0.686	–		
5. DASS_anx	−0.430	−0.425	−0.474	0.653	–	
6. DASS_stress	−0.457	−0.490	−0.525	0.746	0.692	–

For the main analysis, a 2 × 2 mixed-model MANOVA including a Box-M test was conducted in SPSS, with the intervention group as a between-subject factor (SAM vs. education) and time (preintervention vs. postintervention) as a within-subject factor. Interaction effects between these factors and their respective main effects were considered. Partial *η*^2^ was used to indicate the effect size if an effect was significant. In the case of significant multivariate effects, follow-up univariate repeated-measures ANOVAs were performed for each outcome variable to identify the specific variables contributing to the overall effect. To control for multiple testing, Bonferroni correction was applied, resulting in an adjusted significance level of *p* < 0.008 (α_adj_ = 0.05/6). Effect sizes for univariate analyses are reported as partial *η*^2^.

## Results

### Sample description

The baseline assessment was conducted in November 2022. After the three-week intervention period, post-assessment took place in December 2022. There were zero non-completers in the preintervention dataset, but 23 non-completers were observed in the postintervention dataset, which was treated as lost to follow-up in the CONSORT flow diagram (Figure 1). The final sample consisted of *N* = 118 participants who participated in both the baseline and post-assessment. One participant was subsequently excluded from the multivariate analysis because of missing values in one questionnaire at the postintervention assessment. A high attrition rate was observed (73%), which is not uncommon in psychotherapy and related research fields (e.g., Eysenbach, 2005; Melville et al., 2010; Meyerowitz-Katz et al., 2020). However, owing to the high attrition rate, we analysed potential differences between those who discontinued the study (*n* = 324) and those who fully completed the study (*n* = 117). We found no significant group differences regarding gender (*χ^2^*(2) = 0.30, *p* = 0.859), nationality (*χ^2^*(4) = 4.12, *p* = 0.391), group (IG vs. CG; *χ^2^*(1) = 1.92, *p* = 0.165). or age (*t*(439) = −1.02, *p* = 0.307). Furthermore, independent *t* tests revealed no significant group differences in the WHO-5 score (*t*(439) = 0.26, *p* = 0.798), DASS_dep_ score (*t*(439) = 0.85, *p* = 0.396), DASS_anx_ score (*t*(439) = 0.07, *p* = 0.947), or DASS_stress_ score (*t*(439) = 0.28, *p* = 0.781), indicating that no systematic dropout was apparent.

Most of the sample was female (*n* = 97, 82.2%), but a significant proportion was male (*n* = 20, 16.9%), whilst one person identified as nonbinary. The ages ranged from 18 to 61 years, with a mean age of 25.83 years (*SD* = 7.306). In the intervention group, the mean age was 25.53 years (*SD* = 7.075; range: 18–48). The mean age of the control group was slightly higher at 26.08 years (*SD* = 7.534; range: 18–61). More than half of the participants were Austrian (*n* = 66, 55.9%), followed by German (*n* = 34, 28.8%) and Italian (*n* = 10, 8.5%), and the rest (*n* = 8, 6.8%) belonged to other nationalities. In the sample, *n* = 29 (24.6%) lived alone, *n* = 37 (31.4%) shared a flat, *n* = 20 (16.9%) lived with their parents, and *n* = 23 (19.5%) lived with a partner. Some students (*n* = 9, 7.6%) reported not living under any of these circumstances. Descriptive statistics for all six outcome variables are presented in Table 2.

**Table 2:** Descriptive statistics for outcome measures by time and group.

Measure	M (SD)	
	SAM^a^	Control^b^
WHO (t1)	11.77 (4.75)	12.05 (4.21)
WHO (t2)	13.36 (4.92)	13.78 (4.65)
MAAS (t1)	56.70 (12.57)	52.85 (11.10)
MAAS (t2)	57.09 (11.17)	55.03 (10.65)
SCS (t1)	2.88 (0.66)	2.92 (0.55)
SCS (t2)	3.16 (0.63)	3.11 (0.55)
DASS_dep (t1)	6.79 (5.20)	6.37 (4.42)
DASS_dep (t2)	5.64 (5.41)	4.63 (4.75)
DASS_anx (t1)	5.23 (4.41)	4.23 (3.98)
DASS_anx (t2)	3.98 (3.77)	3.32 (3.66)
DASS_str (t1)	8.45 (4.78)	8.02 (4.75)
DASS_str (t2)	6.96 (4.44)	6.48 (4.95)

*N* = 118. ^a^*n* = 53; ^b^*n* = 65. WHO, Well-Being Index; MAAS, Mindful Attention and Awareness Scale; SCS, Self-Compassion Scale; DASS, Depression, Anxiety and Stress Scales; dep., depression subscale; anx., anxiety subscale; str., stress subscale; t1, preintervention assessment; t2, postintervention assessment.

### Preliminary analysis

Inspection of the skewness and kurtosis indicated no deviation from normality, except for one item. Because values were only marginally above the cut-offs and MANOVA procedures were rather robust against violations of normality (Finch, 2005), this deviation was not considered a problem. Levene’s tests also yielded no significant differences between the two groups, suggesting homoscedasticity. Box’s M test also revealed homogenous covariance matrices between groups, with *F*(78, 38849.55) = 0.882, *p* = 0.762. The Mahalanobis distance indicated three possible multivariate outliers, but since the results did not change after exclusion, only the results in which those outliers are included are presented. Detailed descriptive statistics, including skewness and kurtosis, are provided in the Supplementary material. No baseline differences in the outcome variables were detected between the two groups (Online Supplementary material).

### Time and group effects

The mixed MANOVA indicated no significant interaction effect of treatment arm x time, with Pillai’s trace = 0.054, *F*(6, 110) = 1.044, and *p* = 0.401. The main effect on treatment arms was also not significant, with Pillai’s trace = 0.067, *F*(6, 110) = 1.308, and *p* = 0.260, but the main effect on time points was significant, with Pillai’s trace = 0.312, *F*(6, 110) = 8.299, and *p* < 0.001. The effect size for this significant time effect was partial *η*^2^ = 0.312. Follow-up univariate repeated-measures ANOVAs revealed significant main effects of time for self-compassion (SCS; *F*(1,116) = 43.18, *p* < 0.001, *η*^2^ = 0.271), well-being (WHO-5; *F*(1,116) = 15.44, *p* < 0.001, *η*^2^ = 0.117), depression (DASS_dep; *F*(1,116) = 14.70, *p* < 0.001, *η*^2^ = 0.112), anxiety (DASS_anx; *F*(1,116) = 10.32, *p* = 0.002, *η*^2^ = 0.082), and stress (DASS_stress; *F*(1,115) = 13.79, *p* < 0.001, *η*^2^ = 0.107), indicating improvements over time. No significant time effects were observed for mindfulness (MAAS; *F*(1,116) = 2.30, *p* = 0.132, *η*^2^ = 0.019). All significant effects remained after Bonferroni correction. Further, no significant time × group interactions were observed for any outcome (all *p* > 0.05), indicating that changes over time did not differ between the intervention and control group.

Using the established WHO-5 cut-off score (≤15), the proportion of participants classified as having clinically relevant reduced well-being decreased from 36.9 to 29.2% in the control group and from 39.6 to 35.8% in the SAM group. Although these descriptive findings suggest improvements in well-being over time, McNemar tests indicated that the reductions were not statistically significant in either the control group (*p* = 0.332) or the SAM group (*p* = 0.791). Therefore, changes in continuous well-being scores were not accompanied by significant changes in the proportion of participants falling below the clinical cut-off.

Reliable change analyses (RCI) indicated that the proportion of participants showing clinically meaningful improvement varied across outcomes. The highest rates of improvement were observed for well-being (21.2%) and self-compassion (20.3%), whereas lower rates were found for depression (5.9%), anxiety (4.2%), and stress (2.6%). For mindfulness, 13.6% showed clinically meaningful improvement, despite the absence of a significant time effect. Detailed results are presented in Supplementary Table S3. Across the full sample, RCI analyses indicated particularly strong rates of clinically meaningful improvement in well-being and self-compassion, alongside a subset of participants showing reliable deterioration in depression and stress, which is consistent with the absence of a differential SAM effect reported above.

## Discussion

The current study aimed to evaluate the preliminary efficacy of SAM, a newly developed online health promotion intervention for addressing students’ mental health and well-being. The RCT compared this 21-day health promotion programme to a low-dose educational content group using a mixed-model MANOVA. No significant main effect was observed in the treatment group, except for time, thus impeding inferences about the causal mechanisms of SAM. However, as additional factors that may have contributed to these results were not examined, the absence of significant effects should not be interpreted as definitive evidence of programme inefficacy.

One possible explanation for the null between-group findings relates to the absence of a nonactive control group. As the literature indicates that (online) MBIs are effective tools for improving mental health amongst students (Gong et al., 2023; González-Martín et al., 2023; Regehr et al., 2013), the results should be interpreted in light of the lack of an inactive control group. In the current study, both conditions received an intervention, albeit with varying intensity and programme content. Given that university students are considered a high-risk population for mental health issues (Mason et al., 2025; Paiva et al., 2025), we opted for this option, as it seemed more ethically justifiable to us. However, the use of an active comparison condition complicates the interpretation of the findings, as improvements observed over time may reflect shared intervention-related benefits rather than the absence of SAM-specific efficacy. This interpretation is consistent with findings by Alrashdi et al. (2024), who reported no effect of online MBIs compared with an active control group or other treatments; a significant effect was observed when compared with nonactive control groups. In addition, the low-dose educational content control group may itself have produced beneficial effects. Self-guided digital lifestyle interventions addressing behaviours such as sleep, exercise, and nutrition have been associated with small-to-moderate improvements in depression, anxiety, and stress symptoms (Brinsley et al., 2025). Consequently, the present study cannot determine whether the comparable outcomes observed across groups reflect limited efficacy of SAM or beneficial effects shared across both intervention conditions.

Intervention structure and intensity may also have influenced the results. SAM was designed as a highly structured 21-day programme involving daily morning and evening exercises ranging from approximately 5 to 45 min in duration. Compared with other online mindfulness-based interventions, which typically span approximately 8 weeks and involve weekly sessions, the present intervention was relatively brief whilst requiring a comparatively high frequency of exercises as well as self-motivation (Spijkerman et al., 2016). Although this structure was intended to promote regular engagement, the fixed daily format may have reduced flexibility in integrating the exercises into students’ routines. In line with other studies (Fagioli et al., 2023; Hall et al., 2018; Karing and Beelmann, 2021), low-dose MBIs should be sufficient for implementing prospective student intervention programmes. Hall et al. (2018) argued that these interventions can be particularly beneficial for students with high stress levels, as they are reluctant to use traditional counselling services. Given evidence that self-guided mindfulness interventions can effectively be learned through self-help formats and have been shown to contribute to reductions in depression and anxiety (Cavanagh et al., 2014), future iterations of SAM may benefit from allowing participants to adapt the frequency and dosage of exercises more flexible according to their individual needs and schedules. Nevertheless, because the present study did not directly manipulate intervention dosage or duration, these interpretations remain tentative and should be investigated systematically in future research.

Further, a common problem amongst online-based interventions is low adherence and study attrition, which may also have contributed to the findings (Linardon and Fuller-Tyszkiewicz, 2020). Adherence may be especially important in MBIs, as the development of mindfulness skills generally depends on consistent and repeated practise (Carmody and Baer, 2008). Higher engagement has been associated with better outcomes, with studies suggesting a dose–response relationship between practise frequency, session completion, and treatment effects (Cavanagh et al., 2014). Students indicate busy schedules, missing personal contact, or a lack of pressure, such as booked appointments or fees, as reasons for dropping out (Ciharova et al., 2023). Users have also expressed concerns regarding the lack of personalization in online interventions compared with face-to-face therapies (Stawarz et al., 2019). Therefore, personalization processes should be considered in the development of online interventions to enhance user commitment (Oti and Pitt, 2021).

Nevertheless, it is important to note that adherence was not monitored during the implementation period, and the extent to which SAM practises were performed daily remains unknown. To counteract potential nonadherence, regular reminder messages were deliberately sent out, as they demonstrated effectiveness in increasing compliance with various health care behaviours (e.g., Frascella et al., 2020; Wang et al., 2020). Although there is ambiguous evidence that reminders initially increase student adherence, this effect is not sustainable (Hall et al., 2018). In addition, there is a risk that a defensive reaction will be triggered if the individual feels threatened in their free behaviour (Miron and Brehm, 2006), probably because the programme is too tightly meshed. SAM, as a self-administered and rather high-intensity intervention, could have been susceptible to such effects. As a result, participants could have stopped engaging in the intervention content but continued to fill out measures.

The high dropout rate can be explained by (a) time-related conditions, such as exams and pre-Christmas stress, and (b) motivational conditions, such as the high level of effort required for time-consuming exercises and a low level of commitment. In a qualitative study (Lawler et al., 2021), 33% of the participants attributed their dropout to changes in motivation and 53% attributed it to negative reasons, including a divergent mindset or perceiving the iCBT intervention as unsuitable for their individual problem context. A meta-analysis (Karyotaki et al., 2015) on self-guided web-based interventions for depression reported a dropout rate of 59% halfway through the intervention. Further, 11% dropped out before completing 75% of the treatment modules. Finally, only 17% completed all the modules. In general, qualitative data would be informative in explaining the high dropout rate, which should be taken into account in future studies.

The mode of intervention delivery may also have played a role in shaping the observed outcomes. SAM content was delivered exclusively via email, which may have limited usability and participant engagement. University students are typically exposed to high volumes of emails daily, increasing the likelihood that intervention emails are overlooked (Clegg et al., 2026). In addition, email-based delivery may have reduced accessibility and repeated practise, as intervention materials were distributed across multiple messages rather than integrated within a centralised platform. This contrasts with many contemporary online MBIs, which are commonly delivered via websites or smartphone applications (e.g., Gong et al., 2023; Spijkerman et al., 2016). Persuasive design elements (e.g., personalization, self-monitoring, progress tracking, interactive feedback) may improve both adherence and treatment outcomes in digital interventions (Kelders et al., 2012; McCall et al., 2021). However, because user experience and usability were not assessed in the present study, no definitive conclusions can be drawn regarding the impact of delivery format on engagement or outcomes.

Despite our preliminary findings, mindfulness has been identified as a promising approach to improve mental health in the academic setting (Chiodelli et al., 2022; Galante et al., 2018; Sun et al., 2021). Therefore, MBIs such as SAM should continue to be pursued. SAM provides a strong theoretical framework that indicates positive outcomes by including other relevant concepts, such as self-compassion and self-care (e.g., Carvalho et al., 2017; Hayes and Hofmann, 2021). The lack of statistical significance for effects other than time implies that more closely monitored designs, including adherence cheques, are needed to assess whether the true efficacy of SAM has been adequately captured.

As the RCI indicates, the interventions were effective in strengthening positive psychological resources (well-being, self-compassion). However, for a significant proportion of the participants, the interventions were not only ineffective but actually had a negative effect, which could be attributed to potential stress overload (during a period of intensive exams) or other contextual factors.

Nevertheless, the first adaptations could already aim to change the structure (e.g., flexibility and intensity) and the intervention content to increase our health promotion programmes’ overall efficacy, time, and economic efficiency. To date, only the general practises of established MBIs have been incorporated. Nevertheless, transitional stressors such as high demands in the academic setting, social pressure, or unfamiliar environments seem to threaten students’ mental health (Dawson et al., 2020). This suggests that the coverage of student-specific topics may be necessary for understanding their world and, consequently, for achieving sustainable changes. This assumption aligns with that of Bergold and Thomas (2017), who argue that participatory research is crucial to understanding the real world of people affected and involved. Therefore, interventions may not reach their full potential when key life issues are not addressed and processed.

Identifying such population-based issues and the resulting distinct mechanisms of change of MBIs in specific target groups (Alsubaie et al., 2017) needs to be addressed in future research. These questions are also essential in the context of university students to ensure that student-tailored interventions that provide efficient and affordable care are provided. Based on the starting points discussed, SAM is currently under revision to make the best possible contribution to this area of care. However, the transdiagnostic approach of SAM, achieved by mindfulness and ACT (Dawson et al., 2020; Dindo et al., 2017; Gloster et al., 2020), is further maintained to target a broad range of mental health issues whilst concurrently complementing it with approaches for specific stressors.

### Limitations and future directions

It is noteworthy that this RCT has several strengths and limitations. On a positive note, the current study has an RCT design and a nationwide recruitment strategy. However, there have been methodological concerns interfering with the interpretation of the results.

First, adherence cheques and user experience data (perceived helpfulness, usability, willingness to continue) were necessary to conclude treatment success, particularly given the high attrition rate. It remains unclear whether participants consistently received, opened, or engaged with the intervention emails. Some students may not regularly monitor their university email accounts, and it is also possible that intervention messages were filtered into spam or junk folders, thereby limiting exposure to the intervention content. This omission could have led to biassed results, and consequently, implementing monitoring strategies, i.e., tracking technologies, is imperative in future studies. In addition, using qualitative assessments would allow a deeper exploration of barriers to participation and could offer insights into mechanisms of change, going beyond the simple reduction of symptoms. However, the path may be moving more towards using artificial intelligence.

The already mentioned attrition and dropout rates indicate weaknesses in the study design and/or programme structure. Nevertheless, the rates could partially, but not entirely, be explained by other factors. For instance, the timing of the intervention phase was unfavourable, as December is a stressful period for a significant proportion of students because of upcoming exams. Hence, the implementation of exercises might have been additionally challenging because of time constraints and stress, which act as antagonists of a mindful stance.

Furthermore, the lack of pre-registration represents a significant limitation, which does not align with current best practises for RCTs. Pre-registration was unfortunately overlooked at study initiation. Whilst the lack of pre-registration cannot be dismissed, it may be viewed in the context that the reported outcomes correspond to those prespecified in the ethics application approved before participant recruitment.

Given the exploratory nature of the study, no *a priori* power calculation was conducted, as the study aimed to be accessible to all interested students. In addition, baseline data from the full sample are intended for use in a separate part of the project focusing on psychometric analyses based on item response theory (IRT). However, a sensitivity analysis indicated that with *N* = 118, *α* = 0.05, and two measurement time points, the study had 80% power to detect effects of *f*(V) = 0.260 (G*Power 3.1, Faul et al., 2009). The substantial attrition resulted in a reduced final sample size, which may have limited the statistical power to detect effects. Furthermore, due to the high proportion of missing post-intervention data, the application of imputation methods was not considered, as it would have required strong assumptions about the missing data mechanism that may not be fully met and could potentially introduce additional bias. Therefore, we chose to report complete case analyses and explicitly discuss the results with caution.

With respect to the programme content, the exercises might have been too long, resulting in noncompletion, and no transfer of learning to daily life was offered, which might be reflected in the nonsignificance of the results. Future studies should consider crucial times in the academic year as well as students’ time resources when conducting trials.

In addition, the sample was self-selected, with a predominance of female students, which severely limits the generalizability of the findings. Participants who chose to engage in the intervention may have differed systematically from the broader student population with regard to motivation, prior experience with mindfulness practises, attitudes towards mental health, or other personal characteristics that were not assessed or controlled for in the present study. In particular, the underrepresentation of male students is noteworthy, as previous research suggests gender differences in mental health perceptions and help-seeking behaviour (Debate et al., 2022; Gao et al., 2020; Sagar-Ouriaghli et al., 2023). Future adaptations of the intervention should therefore consider the potentially distinct needs, preferences, and barriers to engagement amongst male students.

In conclusion, it should be noted that the theoretical derivation of a relationship between mindfulness, self-compassion, self-care, and well-being served as an initial approach and would need to be further elaborated using a formal mediation model or path diagram.

Building on these limitations, future studies should overcome these shortcomings by implementing the stated recommendations to investigate the programme’s effectiveness.

### Implications

Although no significant between-group effects were observed, mindfulness-based interventions remain a promising approach for improving psychological health in university settings.

## Data Availability

The raw data supporting the conclusions of this article will be made available by the authors, without undue reservation.
