# Supplementary material for: Mindful of yourself: the initial evaluation of an online health promotion programme for university students
Source: Front Psychol. 2026 Jul 20;17:1842551. doi: 10.3389/fpsyg.2026.1842551 (PMC13429853; doi:10.3389/fpsyg.2026.1842551)
Supplement: Supplementary file 1 [file Data_Sheet_1.PDF]

Online Supplemental Material

Mindful of yourself: The initial evaluation of an online health promotion programme for university students

Table S1

SAM Programme Overview

Day	Title	Contents
1	Introducing SAM: Self-Care, Mindfulness, Compassion	In the morning: Pleasure exercise (possibly the "raisin exercise")<br>In the evening: Relaxation exercise (Breathing Space, e.g., https://www.youtube.com/watch?v=JIt5e1zDttY)
2	Mindfulness in Daily Life	Morning: Breathing and Focusing the Mind (e.g., mindful showering)<br>Evening: Guided meditation for beginners (e.g., https://www.youtube.com/watch?v=pT5xvgQ3WxU)
3	… and every beginning holds a certain magic	In the morning: Mindfully confronting negative emotions (Alfred and His Shadow – a short story about emotions https://www.youtube.com/watch?v=igjFCjKwkaM)<br>In the evening: Cooking with a beginner's mindset (stimulating the senses, enjoyment, and gratitude)
4	Living in the Here and Now	In the Morning: Your Safe Place (Guidance on creating your own safe place)<br>In the Evening: Walking Meditation
5	Embrace Slowness	In the Morning: STOP Exercise (Bring your body and mind into balance to reduce stress and anxiety with: S = Stop. T = Take a breath. O = Observe. P = Proceed)<br>In the Evening: Body Scan (e.g., https://www.youtube.com/watch?v=j0nMvbvNcog)
6	Free and self-determined	A longer yoga session for you (e.g., https://www.youtube.com/watch?v=U2OcR9yGzas&list=PL-G7EJFoxFcdpo-f4Z_DuTKkRgRQs5d9t&index=10)
7	Yoga experience	In the Morning: Sitting meditation (e.g., https://www.youtube.com/watch?v=vEmwUoE5dPo&list=PL7MNI2xLic18ViAzQ6DXxc-Q7DYjyuFHF&index=4<br>In the Evening: Mindfulness yoga (e.g., https://www.youtube.com/watch?v=acq30zvNu9Y
8	People are creatures of habit	In the morning: Observe your habits<br>In the evening: Reflect on your habits (You could also ask yourself the following questions: What habits and rituals do I notice in myself? Which ones are helpful and give me structure? Do I feel like I'm on a hamster wheel? Which habit loop would I like to break out of?)
9	Life isn't a walk in the park	In the Morning: SAFE exercise (Explore yourself with SAFE: S = Soften around a strong emotion that is present. A = Allow or acknowledge that it's there. F = Feel in the body

		where the emotion resides. E = Expand this friendly wish to all other people who are experiencing this same feeling right now.<br>In the Evening: Gratitude exercise (Focus on the people and things that enrich your life and for which you are grateful)
10	The Ups and Downs of Life	In the Morning: Hero of the Day<br>In the Evening: Progressive Muscle Relaxation (Quick Guide)
11	Be with me	As I breathe in, I bring peace to my body.<br>As I breathe out, I smile. I stay in the present moment and know that it is a wonderful moment. (Thich Nhat Hanh, 2007)
12	Towards a Mindful Approach to Ourselves	In the Morning: Reflect on Work-Life Balance<br>In the Evening: See Things Through a Friend's Eyes (Practice Self-Compassion)
13	On Approaching Our Mistakes and Imperfections with Compassion	In the Morning: Reflection on Dealing with Mistakes and Self-Criticism<br>In the Evening: Breathing Exercise
14	Joy through Connection	In the Morning: Focus on the pleasant things in life (list of activities)<br>In the Evening: Mindful listening
15	Strength lies in stillness	Enjoy the Day
16	Connection	In the Morning: Treating others with kindness (feeling connected to the world and its beings)<br>In the Evening: Mindful eating and enjoyment (appreciating the process of food production, gratitude)
17	The Opposites of Activity and Rest	In the Morning: Mindfulness Exercise (moving around, such as stretching, running, yoga, qigong, tai chi, swimming, snorkeling, water skiing, scuba diving, ice skating, hang gliding, cycling, …)<br>In the Evening: Time to do nothing
18	Dealing compassionately with negative emotions	In the Morning: Recognizing resistance and negative feelings (oscillating between acceptance and change)<br>In the Evening: Reflecting on a compassionate and mindful attitude
19	Baggy look	Zieh dir an, was dir gefällt. Bequem, flexibel, anpassungsfähig - ein Kleidungsstück, das dir Entspannung und Entschleunigung bringt.<br>Wear whatever you like. Comfortable, flexible, adaptable—a piece of clothing that helps you relax and slow down.
20	Finding Direction in Life	In the morning: Create a values compass and act in accordance with your values<br>In the evening: Set specific goals using the SMART approach
21	Phoenix Rising	Morning: Ray of Hope Exercise (self-compassion to help you overcome challenges)<br>Evening: A walk (be open to new experiences; engage all your senses)

Table S2

Examples of motivational emails

Day	Content
**7**	Great, you've already made it through a week—keep it up.
**11**	Live in the here and now - stay focused and practice mindfulness.
**14**	Remember to treat yourself like your best friend!
**17**	Find joy in life's little things - you are valuable!
**21**	Your body works hard - be grateful!

Table S3

Reliable Change Indices (RCI) for all six outcome variables.

Measure	Improvement (%)	No change (%)	Deterioration (%)
WHO-5	21.2	72.9	5.9
SCS	20.3	75.4	4.2
DASS-Dep	5.9	74.6	19.5
DASS-Anx	4.2	88.1	7.6
DASS-Stress	2.6	83.8	13.7
MAAS	13.6	79.7	6.8

Note. Values represent the percentage of participants showing reliable improvement ($RCI > 1.96$), no reliable change ($-1.96 \leq RCI \leq 1.96$), or reliable deterioration ($RCI < -1.96$), based on the method by Jacobson and Truax (1991).

References

Jacobson, N. S., & Truax, P. (1991). Clinical significance: A statistical approach to defining meaningful change in psychotherapy research. *Journal of Consulting and Clinical Psychology*, *59*(1), 12–19. https://doi.org/10.1037/0022-006X.59.1.12
